# The clinical features and prognosis of patients with mucinous breast carcinoma compared with those with infiltrating ductal carcinoma: a population-based study

**DOI:** 10.1186/s12885-021-08262-0

**Published:** 2021-05-11

**Authors:** Xingtong Zhou, Zhibo Zheng, Yan Li, Weiwei Zhao, Yan Lin, Jieshi Zhang, Qiang Sun

**Affiliations:** 1 Department of Breast Surgery, Peking Union Medical College Hospital, Chinese Academy of Medical Sciences, No 41 Damucang Hutong, Xicheng District, Beijing, 100032 China; 2 Department of International Medical Services, Peking Union Medical College Hospital, Chinese Academy of Medical Sciences, Beijing, China; 3 Department of Epidemiology and Biostatistics, School of Public Health, Harbin Medical University, Harbin, China

**Keywords:** Mucinous breast carcinoma, Surveillance, epidemiology, and end results database, Clinical features, Prognosis

## Abstract

**Background:**

At present, the characteristics of mucinous breast carcinoma (MBC) and the factors affecting its prognosis are controversial. We compared the clinical features of MBC with those of infiltrating ductal carcinoma (IDC) and summarized the relevant prognostic factors.

**Methods:**

The Surveillance, Epidemiology, and End Results (SEER) database includes information on 10,593 patients diagnosed with MBC between 2004 and 2016. Chi-square tests and analyses were used to analyze differences in variables between the MBC and IDC groups. Univariate and multivariate Cox proportional hazards models were used to assess the relative impacts of risk factors on cancer-specific survival (CSS) in patients. Kaplan-Meier survival curves were constructed to assess cancer-specific mortality and were compared using the log-rank test.

**Results:**

From 2004 to 2016, 10,593 people were diagnosed with MBC, and 402,797 were diagnosed with IDC. Patients with MBC had significantly higher 5−/10-year CSS rates (96.4%/93.4%) than those with IDC (89%/83.8%). Compared with IDC patients, MBC patients had less lymph node metastasis, an earlier stage, a higher rate of hormone receptor positivity and a lower expression rate of HER2. Univariate and multivariate analyses showed that age ≥ 60 years old (HR = 1.574, 95%CI: 1.238–2.001, *P* < 0.001), singled status (HR = 1.676, 95%CI: 1.330–2.112, *P* < 0.001) and advanced TNM/SEER stage were independent prognostic risk factors for MBC. In addition, positive estrogen receptor (HR = 0.577, 95%CI: 0.334–0.997, *P* = 0.049), positive progesterone receptor (HR = 0.740, 95%CI: 0.552–0.992, *P* = 0.044), surgical treatment (HR = 0.395, 95%CI: 0.288–0.542, *P* < 0.001) and radiotherapy (HR = 0.589, 95%CI: 0.459–0.756, *P* < 0.001) were identified as protective factors.

**Conclusion:**

Compared with IDC, MBC has a better prognosis. For patients with MBC, we identified prognostic factors that can help clinicians better assess patient outcomes and guide individualized treatment.

## Background

Breast carcinoma is the most common cancer and leading cause of death among women worldwide. Infiltrating breast carcinoma accounts for the vast majority of all breast cancer types. Infiltrating ductal carcinoma (IDC) is the most common subtype of infiltrating breast cancer, accounting for approximately 70–80%, whereas mucinous breast carcinoma (MBC) is a rare and special subtype.

Currently, MBC is subdivided into simple mucinous carcinoma and mixed mucinous carcinoma based on whether it contains other types of tumor components. The pathology of simple mucinous carcinoma of the mammary gland is characterized by the cluster-like hyperplasia of tumor cells floating in extracellular mucous fluid, and mucinous cancer components account for more than 90% of all tumor cells [1]. In addition to mucinous components, mixed mucinous carcinoma also contains in situ ductal carcinoma or other invasive carcinoma components. It is reported that MBC accounts for 1–6% of all breast carcinoma and approximately 2.4% of all infiltrating breast carcinoma [2, 3]. MBC is common among postmenopausal women, and its clinical features are different from those of IDC. High expression of hormone receptors and low expression of human epidermal growth factor receptors (e.g., HER2) were also observed [4–6]. Moreover, the prognosis of MBC patients has been shown to be better than that of IDC patients [6]. The incidence of recurrence or distant metastasis in typical simple MBC patients is low. Most MBC patients receive postoperative adjuvant endocrine therapy, and fewer patients with MBC need chemotherapy and radiotherapy compared with those with other types of breast cancer [7].

Breast cancer is characterized as a highly heterogeneous tumor, and many clinical features may be prognostic factors for patients. As a rare tumor, MBC has a good prognosis, but its clinical features and prognostic factors are still controversial. The aim of this study was to compare the pathogenesis, clinical features and prognosis of MBC with those of IDC by a statistical analysis based on the Surveillance, Epidemiology, and End Results (SEER) database. We also evaluated the impact of clinical features on survival in MBC patients, further to identify the prognostic factors associated with cancer-specific survival (CSS).

## Methods

### Participants

The data used in this study were obtained from the SEER database, which is developed by the National Cancer Institute (NCI). The SEER database contains epidemiological characteristics, primary tumor characteristics, progression stages, treatment options and follow-up information of various malignancies, covering approximately 34.6% of the population in the United States [8]. SEER*Stat 8.3.6 software was used to extract information from the database. We screened information on patients diagnosed with breast cancer January 1, 2004 to December 31, 2016. The pathological diagnosis codes were 8500/3 (IDC) and 8480/3 (MBC). Due to the openness and availability of SEER data, our study was deemed exempt from institutional review board approval.

The exclusion criteria were as follows: 1) patients who lacked major information (e.g., age, tumor pathological type, follow-up information, cause of death); 2) patients with other malignancies found at diagnosis or during the follow-up period (patients with MBC had a higher survival rate than patients with other malignancies; thus, these data would affect CSS if patients with other primary tumors were incorporated into the study). In this study, the following data of MBC and IDC patients were extracted from the SEER database: gender, age, race, marital status, tumor location, grade, stage, estrogen receptor (ER) status, progesterone receptor (PR) status, human epidermal growth factor receptor 2 (HER2) status, treatment history and follow-up information. Additional comparisons of MBC and IDC patients were also performed.

### Statistical analysis

All statistical analyses were conducted by SPSS 25.0 (SPSS Inc., Chicago, IL, USA) and GraphPad Prism 7 (GraphPad Software). Clinical information and tumor features were summarized with descriptive statistics. Comparisons of categorical variables among different groups were performed by using the Chi square test. Univariate and multivariate Cox proportional hazards models were used to assess the relative impacts of risk factors for CSS in patients. Kaplan-Meier survival curves were constructed to assess cancer-specific mortality, and their comparisons were conducted by using the log-rank test. *P* < 0.05 was considered statistically significant.

## Results

### Overview of MBC and IDC patients

From 2004 to 2016, a total of 10,593 people were diagnosed with MBC, and 402,797 were diagnosed with IDC. The median age of MBC patients was 68 years old (ranged 21–105 years old), and the median follow-up period was 60 months (ranged 1–155 months). The median age of IDC patients was 59 years old (range 15–118 years old), and the median follow-up period was 53 months (ranged 1–155 months). In this study, CSS was defined as endpoint. MBC patients’ 5−/10-year CSS rates were 96.4%/93.4%, while IDC patients’ 5−/10-year CSS rates were 89%/83.8% (*P* < 0.001).

### Comparison of baseline characteristics between MBC and IDC patients

The epidemiologic features, clinical features, tumor stage, and pathological features of MBC and IDC patients are summarized in Table 1. In both MBC and IDC patients, men accounted for a very small percentage (0.4 and 0.8%, respectively). Besides, the age of women ≥60 years old accounted for 68.0% of MBC patients and only 48.3% of IDC patients (*P* < 0.001). In terms of the location of the tumor, it was more common in the upper-outer quadrant of the breast, with 50.2% in MBC and 57.1% in IDC (Table 1).

**Table 1:** Patients characteristics of MBC patients and IDC patients

Characteristics	MBC *N* = 10,593 (%)	IDC *N* = 402,797 (%)	*P*
Sex			< 0.001
Female	10,552 (99.6)	399,699 (99.2)	
Male	41 (0.4)	3098 (0.8)	
Age			< 0.001
< 60 years old	3395 (32.0)	208,149 (51.7)	
≥ 60 years old	7198 (68.0)	194,648 (48.3)	
Race			< 0.001
White	8102 (76.5)	315,613 (78.4)	
Black	1177 (11.1)	45,881 (11.4)	
Other	1314 (12.4)	41,303 (10.3)	
Marital status			< 0.001
Married	4993 (47.1)	224,449 (55.7)	
Singled	5054 (47.7)	158,735 (39.4)	
Unknown	546 (5.2)	19,613 (4.9)	
Location			< 0.001
Central portion of breast	847 (8.0)	21,247 (5.3)	
Upper-inner quadrant	1232 (11.6)	48,162 (12.0)	
Lower-inner quadrant	977 (9.2)	22,735 (5.6)	
Upper-outer quadrant	5318 (50.2)	229,970 (57.1)	
Lower-outer quadrant	938 (8.9)	29,175 (7.2)	
Unspecific	1281 (12.1)	51,508 (12.8)	
Grade			< 0.001
I	5582 (52.7)	74,859 (18.6)	
II	3335 (31.5)	159,214 (39.5)	
III	389 (3.7)	150,305 (37.3)	
IV	21 (0.2)	2554 (0.6)	
Unspecific	1266 (12.0)	15,865 (3.9)	
SEER stage			< 0.001
Local	9134 (86.2)	252,467 (62.7)	
Regional	1137 (10.7)	127,795 (31.7)	
Distant	197 (1.9)	18,965 (4.7)	
Unspecific	125 (1.2)	3570 (0.9)	
T-stage			< 0.001
T1	6732 (63.5)	234,608 (58.2)	
T2	2816 (26.6)	119,558 (29.7)	
T3	519 (4.9)	20,073 (5.0)	
T4	207 (2.0)	16,860 (4.2)	
Unspecific	320 (3.0)	11,698 (2.9)	
N-stage			< 0.001
N0	9058 (85.5)	255,082 (63.3)	
N1	822 (7.8)	93,750 (23.3)	
N2	143 (1.3)	22,938 (5.7)	
N3	99 (0.9)	15,704 (3.9)	
Unspecific	471 (4.4)	15,323 (3.8)	
M-stage			< 0.001
M0	10,202 (96.3)	379,350 (94.2)	
M1	183 (1.7)	17,192 (4.3)	
Unspecific	208 (2.0)	6255 (1.6)	
TNM stage			< 0.001
I	6381 (60.2)	187,086 (46.4)	
II	3183 (30.0)	139,311 (34.6)	
III	402 (3.8)	45,608 (11.3)	
IV	183 (1.7)	17,195 (4.3)	
Unspecific	444 (4.2)	13,597 (3.4)	
ER			< 0.001
Negative	187 (1.8)	83,543 (20.7)	
Positive	9915 (93.6)	305,234 (75.8)	
Unspecific	491 (4.6)	14,020 (3.5)	
PR			< 0.001
Negative	1036 (9.8)	123,454 (30.6)	
Positive	8958 (84.6)	262,619 (65.2)	
Unspecific	599 (5.7)	16,724 (4.2)	
HER2			< 0.001
Negative	5124 (48.4)	182,050 (45.2)	
Positive	286 (2.7)	40,845 (10.1)	
Unspecific	5183 (48.9)	179,902 (44.7)	
Molecular subtypes			< 0.001
Luminal A	5088 (48.0)	153,463 (38.1)	
Luminal B	255 (2.4)	28,240 (7.0)	
HER2-enriched	30 (0.3)	12,517 (3.1)	
Basal subtypes	29 (0.3)	28,371 (7.0)	
Unspecific	5191 (49.0)	180,206 (44.7)	
Surgery			< 0.001
No	580 (5.5)	25,525 (6.3)	
Yes	9952 (93.9)	373,914 (92.8)	
Partial mastectomy	6713 (63.4)	222,175 (55.2)	
Total mastectomy^a^	1878 (17.7)	75,800 (18.8)	
Radical mastectomy^b^	1334 (12.6)	74,648 (18.5)	
Unknown	27 (0.3)	1291 (0.3)	
Unspecific	61 (0.6)	3358 (0.8)	
Radiotherapy			< 0.001
No	5530 (52.2)	196,512 (48.8)	
Yes	5063 (47.8)	206,285 (51.2)	
Survival			< 0.001
5-year CSS rate	96.4	89.0	
10-year CSS rate	93.4	83.8	
Early stage 5-year CSS rate	97.67	92.00	
Advanced stage 5-year CSS rate	38.81	27.70	

^a^A simple mastectomy removes all breast tissue, the nipple, and areolar complex. An axillary dissection is not done

^b^Radical mastectomy includes modified radical mastectomy, radical mastectomy NOS and extended radical mastectomy

### Comparison of pathological characteristics between MBC and IDC patients

Compared with IDC, MBC was characterized by lower lymph node metastasis rate, earlier stage, higher expression rate of ER and PR, and lower expression rate of HER2. In MBC, low-grade tumors accounted for 84.2% (grade I 52.7%, grade II 31.5%), while in IDC, grade I-III accounted for 18.6, 39.5 and 37.3%, respectively. At the time of MBC diagnosis, 85.5% of patients were in the N0 stage; while at the time of IDC diagnosis, 63.3% of patients were in the N0 stage. Among the MBC patients, 86.2% were in the local stage and 10.7% were in the regional stage; while among the IDC patients, 62.7% were in the local stage and 31.7% were in the regional stage. Immunohistochemical analysis of MBC tumors showed that the ER-positive rate was 93.6%, the PR-positive rate was 84.6%, and the HER2-positive rate was 2.7%; while in IDC tumors, the ER-positive rate was 75.8%, the PR-positive rate was 65.2%, and the HER2-positive rate was 10.1%. In total, 48% of MBC patients had the Luminal A subtype, and 0.3% had the basal-like subtype. However, 38.1% of IDC patients had the Luminal A subtype, and 7.0% had the basal-like subtype (Table 1).

### Comparison of treatment between MBC and IDC patients

The vast majority of MBC and IDC patients underwent surgery (93.9% with MBC and 92.8% with IDC). A total of 47.8% of MBC patients received postoperative radiotherapy compared with 51.2% of IDC patients (Table 1).

### Survival analysis of MBC patients

**Fig. 1:**
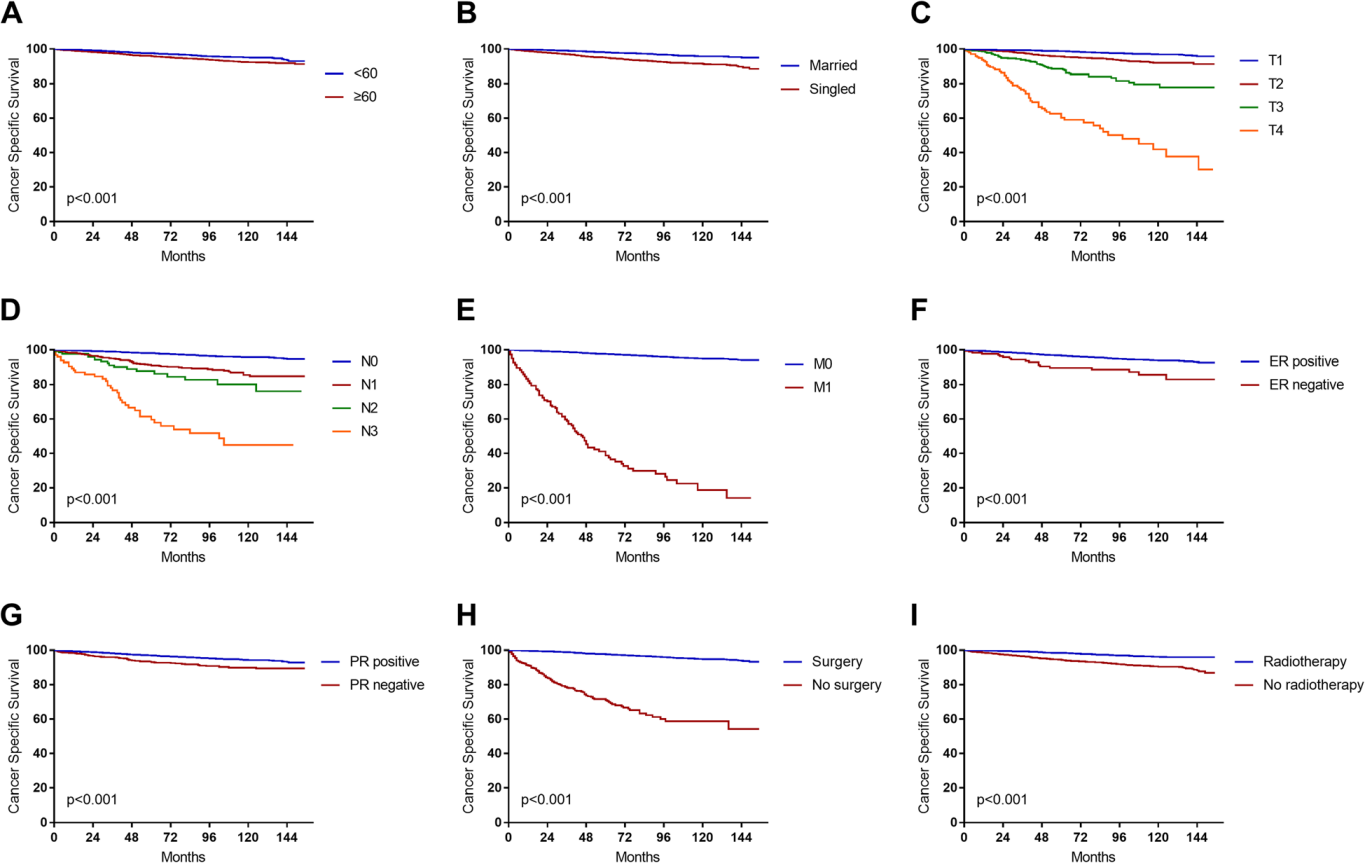
The cancer-specific survival of patients with MBC associated with different factors. **a** Age < 60 and Age ≥ 60; **b** Marital status; **c** T stage; **d** N stage; **e** M stage; **f** ER; **g** PR; **h** Surgery; **i** Radiotherapy

We also analyzed the risk factors for CSS in MBC patients by using the Cox regression model. Multivariate analysis showed that age ≥ 60 years old (HR = 1.574, 95%CI: 1.238–2.001, *P* < 0.001), singled status (HR = 1.676, 95%CI: 1.330–2.112, *P* < 0.001) and advanced TNM/SEER stage were independent prognostic risk factors for MBC. In addition, positive estrogen receptor (HR = 0.577, 95%CI: 0.334–0.997, *P* = 0.049), positive progesterone receptor (HR = 0.740, 95%CI: 0.552–0.992, *P* = 0.044), surgery (HR = 0.395, 95%CI: 0.288–0.542, *P* < 0.001) and radiotherapy (HR = 0.589, 95%CI: 0.459–0.756, *P* < 0.001) were identified as protective factors. There was no significant difference in the status of HER2 receptors (*P*>0.05) (Table 2). The CSS estimates were classified by age, marital status, T stage, N stage, M stage, ER, PR, surgery and radiotherapy (Fig. 1).

**Table 2:** Univariate and multivariate analysis of CSS in MBC patients

Features	Univariate		Multivariate	
	HR (95%CI)	*P* value	HR (95%CI)	*P* value
Sex				
Female	1		–	–
Male	1.834 (0.589–5.712)	0.295	–	–
Age				
< 60 years old	1		1	
≥ 60 years old	1.570 (1.256–1.962)	< 0.001	1.596 (1.257–2.025)	< 0.001
Race				
White	1		1	
Black	1.718 (1.325–2.226)	< 0.001	1.201 (0.918–1.572)	0.181
Other	0.462 (0.302–0.706)	< 0.001	0.433 (0.281–0.666)	< 0.001
Marital status				
Married	1		1	
Singled	2.448 (1.964–3.052)	< 0.001	1.679 (1.333–2.114)	< 0.001
Location				
Central portion of breast	1		–	–
Upper-inner quadrant	1.109 (0.691–1.781)	0.667	–	–
Lower-inner quadrant	0.677 (0.389–1.178)	0.168	–	–
Upper-outer quadrant	0.914 (0.615–1.358)	0.656	–	–
Lower-outer quadrant	0.754 (0.436–1.304)	0.313	–	–
Unspecific	3.152 (2.108–4.714)	< 0.001	–	–
Grade				
I	1		–	–
II	1.310 (1.031–1.665)	0.027	–	–
III	2.825 (1.931–4.133)	< 0.001	–	–
IV	2.564 (0.635–10.345)	0.186	–	–
SEER stage				
Local	1		–	–
Regional	4.427 (3.453–5.676)	< 0.001	–	–
Distant	48.018 (37.616–61.296)	< 0.001	–	–
T-stage				
T1	1		1	
T2	2.680 (2.070–3.469)	< 0.001	1.971 (1.499–2.592)	< 0.001
T3	7.896 (5.726–10.889)	< 0.001	3.321 (2.279–4.838)	< 0.001
T4	30.059 (22.181–40.735)	< 0.001	4.382 (2.835–6.773)	< 0.001
N-stage				
N0	1		1	
N1	3.790 (2.881–4.985)	< 0.001	1.642 (1.194–2.256)	0.002
N2	6.135 (3.789–9.932)	< 0.001	2.227 (1.316–3.769)	0.003
N3	21.156 (14.901–30.036)	< 0.001	1.678 (1.052–2.674)	0.030
M-stage				
M0	1		1	
M1	36.539 (28.835–46.302)	< 0.001	6.674 (4.771–9.336)	< 0.001
TNM stage				
I	1		–	–
II	2.967 (2.246–3.920)	< 0.001	–	–
III	10.877 (7.662–15.440)	< 0.001	–	–
IV	75.573 (56.325–101.399)	< 0.001	–	–
ER				
Negative	1		1	
Positive	0.372 (0.234–0.591)	< 0.001	0.559 (0.328–0.953)	0.033
PR				
Negative	1		1	
Positive	0.498 (0.385–0.645)	< 0.001	0.730 (0.545–0.97)	0.034
HER2				
Negative	1		–	–
Positive	0.833 (0.339–2.046)	0.691	–	–
Surgery				
No	1		1	
Yes				
Partial mastectomy	0.047 (0.037–0.061)	< 0.001	0.334 (0.229–0.488)	< 0.001
Total mastectomy^a^	0.095 (0.070–0.129)	< 0.001	0.435 (0.299–0.631)	< 0.001
Radical mastectomy^b^	0.148 (0.113–0.195)	< 0.001	0.470 (0.333–0.665)	< 0.001
Radiotherapy				
No	1		1	
Yes	0.316 (0.252–0.397)	< 0.001	0.668 (0.508–0.880)	0.004

^a^A simple mastectomy removes all breast tissue, the nipple, and areolar complex. An axillary dissection is not done

^b^Radical mastectomy includes modified radical mastectomy, radical mastectomy NOS and extended radical mastectomy

## Discussion

In recent years, the incidence of breast cancer is increasing annually, but the mortality rate is decreasing due to a deeper understanding of breast cancer features and more effective postoperative adjuvant treatment. Breast cancer has a high degree of heterogeneity, and includes different histological types and different molecular subtypes. However, the biological characteristics and clinical outcomes of these subtypes are different [9]. MBC is a special type of invasive breast cancer. Because MBC is rare, there are few studies on this topic, most of which are single-center retrospective studies involving a small sample size of patients. Thus, the purpose of this study was to collect relevant information from the SEER database, and compare the characteristics of MBC with those of IDC, further to determine the risk factors that affect the prognosis of MBC patients.

MBC cells generally express high levels of MUC2 and MUC6. MBC cells secrete mucin and produce a large amount of mucin outside the cell. Cancer cells float as a single or small mass in the mucous and are unable to contact the interstitium, thus reducing their invasiveness. Therefore, in general, metastasis does not occur in early stage of MBC, and the prognosis of MBC has been shown to be better than that of other types of invasive breast cancer [10, 11]. In this study, more patients with MBC were at a low TNM/SEER stage at the time of diagnosis compared with patients with IDC. Consistent with previous findings, the data from this study also confirmed that patients with MBC had significantly fewer lymph node metastases and that the 5−/10-year CSS rates were significantly higher compared with those patients with IDC [12–14]. In addition, older postmenopausal women may be more likely to develop MBC. Among the patients included in this study, the median age of IDC and MBC patients was 59 years old and 68 years old respectively, and there was a significant difference.

Compared with IDC, MBC was better reflected in the immunohistochemistry results: ER and PR positivity and HER2 negativity (*P* < 0.001). Regarding the molecular subtype, most MBC patients have the Luminal A subtype, and the other three types were statistically uncommon in MBC patients compared with IDC patients. Most studies have shown that the expression of hormone receptors is significantly higher in MBC than that in IDC, indicating that MBC is a strong hormone-dependent tumor [15, 16]. The vast majority of MBC patients have the opportunity to receive adjuvant endocrine therapy, thus reducing the risk of local recurrence and distant metastasis after the operation, and the prognosis is significantly better than that of IDC [17, 18]. In addition, the multivariate survival analysis for MBC patients showed that there were significant differences in positive ER and positive PR, suggesting that the prognosis of MBC patients with hormone receptor positivity is better than that of MBC patients with hormone receptor negativity. Furthermore, HER2 overexpression is generally believed to be associated with breast cancer recurrence and metastasis. However, this study did not identify HER2 overexpression as an independent risk factor for prognosis in MBC patients (*P* = 0.083) perhaps due to the gradual widespread use of Herceptin therapy in recent years, improving the prognosis of patients with HER2-positive breast cancer.

TNM stage was revealed as an independent risk factor for prognosis in MBC patients. The later the stage is, the worse the prognosis is. Lymph node involvement has always been considered as an important factor affecting the prognosis of MBC patients [19, 20]. However, whether tumor size is an independent risk factor remains controversial. Some studies have suggested that MBC is mainly composed of mucin, but there is no significant relationship between tumor size and prognosis [21]. However, our data showed that T stage was related to prognosis, and the larger the stage is, the worse the prognosis is. The marital status of the patient also affected the prognosis, we found that a single status was identified as an independent risk factor. Breast cancer patients are overwhelmingly female, and they need emotional support. Married women are more likely to receive psychological support compared with those single women, and mortality of single women was higher than that of married women [22, 23].

We found that receiving surgery and postoperative radiotherapy were important protective factors for MBC patients. Although MBC has a good prognosis, it still needs to follow the guidelines for surgical treatment and postoperative radiotherapy. Considering the low rate of lymph node metastasis in MBC patients, we believe that sentinel lymph node biopsy should be sufficient in the absence of evidence of clinical lymph node metastasis [24]. If the sentinel lymph node is positive, axillary lymph node dissection should be performed again.

There are some limitations in this study. Firstly, for breast tumors, the molecular subtype is an important indicator of prognosis. In the current SEER database, 48.9% of HER2 information is missing. Secondly, Ki67 and P53 are also related to MBC tumor cell proliferation, recurrence and metastasis, and more research about them should be explored in the future [25, 26]. The SEER database does not yet contain this information. We hope that the SEER database will include more details so we can obtain more accurate research results.

## Conclusion

Compared with IDC, MBC is more likely to occur in older female patients, with an earlier tumor stage, a higher positive rate of hormone receptors, a lower positive rate of HER2 and a better prognosis. For patients with MBC, age ≥ 60 years old, single status, and late TNM stage are independent prognostic risk factors, while hormone receptor positivity, surgery and radiotherapy are prognostic protection factors. The HER2 status does not affect prognostic outcomes.

## Data Availability

The SEER database is supported by the National Cancer Institute (www.seer.cancer.gov) and contains a large amount of information, which provides strong data support for the in-depth study of tumors. Our study also used the data provided by the SEER database to analyze and research the clinical features of breast cancer patients. The datasets generated and analyzed during the current study are available from the corresponding author on reasonable request.

## Abbreviations

MBC: Mucinous breast carcinoma
IDC: Infiltrating ductal carcinoma
SEER: The Surveillance, Epidemiology, and End Results
CSS: Cancer-specific survival
NCI: National Cancer Institute
ER: Estrogen receptor
HER2: Epidermal growth factor receptor 2
PR: Progesterone receptor
